# *Fasciola hepatica*‐Derived Proteins Shield the Heart From Type 2 Myocardial Infarction in Rats by Modulating Oxidative Stress and Inflammatory Imbalance: Insights Relevant to the Hygiene Hypothesis

**DOI:** 10.1155/omcl/3759583

**Published:** 2026-03-13

**Authors:** Mohammadreza Ahmadi-Beni, Kobra Mokhtarian, Gholam Reza Mobini, Somayeh Najafi-Chaleshtori, Najmeh Salehi-Vanani, Maryam Anjomshoa, Fariba Houshmand

**Affiliations:** ^1^ Student Research Committee, Shahrekord University of Medical Sciences, Shahrekord, Iran, skums.ac.ir; ^2^ Cellular and Molecular Research Center, Basic Health Sciences Institute, Shahrekord University of Medical Sciences, Shahrekord, Iran, skums.ac.ir; ^3^ Clinical Biochemistry Research Center, Basic Health Sciences Institute, Shahrekord University of Medical Sciences, Shahrekord, Iran, skums.ac.ir; ^4^ Department of Tissue Engineering and Applied Cell Sciences, School of Advanced Technologies, Medical Plants Research Center, Shahrekord University of Medical Sciences, Shahrekord, Iran, skums.ac.ir; ^5^ Department of Anatomical Sciences and Histology, School of Medicine, Shahrekord University of Medical Sciences, Shahrekord, Iran, skums.ac.ir

**Keywords:** *Fasciola hepatica*, hygiene hypothesis, isoproterenol, myocardial infarction, preconditioning

## Abstract

Myocardial infarction (MI) remains a leading cause of mortality worldwide, with type 2 MI (T2MI) carrying a worse prognosis than type 1 MI (T1MI). The hygiene hypothesis suggests that reduced microbial exposure in sanitized environments contributes to immune dysregulation and inflammation‐related diseases. While helminth therapy has shown potential in modulating the inflammatory responses in myocardial injury, its effects on oxidative stress remain underexplored. We hypothesize that *Fasciola hepatica* total protein extract (FhTE) attenuates myocardial injury in T2MI via immune modulation consistent with the hygiene hypothesis, affecting both inflammation and oxidative stress. To investigate this, male Wistar rats were pretreated with FhTE (2.5 mg/kg, intraperitoneally) daily for 6 days. MI was induced by subcutaneous isoproterenol (100 mg/kg) on days five and six. Electrocardiographic analysis 24 h post‐final treatment revealed that FhTE pretreatment attenuated MI‐induced changes. FhTE reduced cardiac hypertrophy and decreased serum cardiac injury markers. It enhanced antioxidant defense by increasing superoxide dismutase (SOD) and catalase (CAT) activities, lowering nitric oxide (NO) and malondialdehyde (MDA) levels, and modulating nuclear factor erythroid 2‐related factor 2 (Nrf2) mRNA levels. FhTE also reduced neutrophil and M1 macrophage activity, evidenced by decreased myeloperoxidase (MPO) levels and inducible nitric oxide synthase (iNOS) mRNA expression, and downregulated inflammatory cytokine genes (IL‐1β, IL‐6, TNF‐⍺, and IL‐33). FhTE demonstrates significant cardioprotective effects by modulating inflammation and oxidative stress, thereby preconditioning the myocardium against T2MI. These findings offer robust experimental support for the hygiene hypothesis in the context of ischemic heart disease, highlighting its potential for novel MI therapies.

## 1. Introduction

Ischemic heart diseases (IHD) are the leading cause of mortality worldwide [1]. Among IHD, myocardial infarction (MI) accounts for the most deaths [2]. According to the Fourth Universal Definition of MI, type 2 MI (T2MI) occurs due to an acute imbalance of myocardial oxygen supply and demand in the context of an acute illness causing tachyarrhythmia, hypoxia, or hypotension without acute atherothrombosis [3]. The incidence of T2MI is similar to type 1 MI (T1MI), which is caused by acute atherothrombotic events, but it is associated with a worse prognosis compared to T1MI [4]. Acute beta‐adrenergic stimulation, triggered by stress or pharmacological agents like isoproterenol (ISO), increases heart rate (HR) and myocardial contractility. These changes elevate the heart’s oxygen demand [5]. When the oxygen supply cannot meet this heightened demand, it results in myocardial ischemia and potentially T2MI [4, 6].

The inflammatory response plays a crucial role in the expansion of damage during T2MI [7]. Following MI, the ischemic injury triggers a cascade of inflammatory events, including the activation of the complement system and the release of pro‐inflammatory cytokines [8, 9]. These cytokines recruit immunocytes to the infarcted area, where they release proteolytic enzymes and reactive oxygen species (ROS) to clear necrotic tissue. However, this process can inadvertently damage surrounding viable myocardium, exacerbating the infarct size [9, 10].

The hygiene hypothesis suggests that reduced exposure to certain microorganisms, such as gut flora and helminth parasites, due to improved hygiene and sanitation practices, has contributed to the rise in autoimmune and allergic diseases [11, 12]. Early exposure to a diverse range of microbes helps properly in training the immune system, promoting tolerance, and preventing overactive immune responses [13]. Recent research has extended this hypothesis to cardiovascular and other organic diseases [14]. The hypothesis posits that early microbial exposure is crucial for developing a balanced immune system, which can help reduce chronic inflammation—a key factor in cardiovascular conditions like atherosclerosis [15, 16]. For instance, studies on populations with high infection burdens, such as the Tsimane people in the Bolivian Amazon, show low rates of coronary artery disease despite high levels of inflammation markers. This suggests that their immune systems might be better at regulating inflammation, potentially due to constant exposure to various pathogens [17].

Helminth‐derived proteins have shown significant potential in reducing acute inflammation and oxidative stress. These proteins modulate the host’s immune response by lowering the levels of pro‐inflammatory cytokines like tumor necrosis factor‐alpha (TNF‐α), interleukin‐1 beta (IL‐1β), and IL‐6 [18]. Additionally, helminth‐derived proteins enhance the activity of antioxidant enzymes, thereby reducing the levels of ROS and mitigating oxidative stress [19]. The total protein extract of *Fasciola hepatica* (FhTE) is a complex mixture derived from the liver fluke, *F. hepatica*. This extract contains a variety of proteins and molecules that have immunomodulatory and antioxidant properties. It can suppress the activation of dendritic cells, reducing pro‐inflammatory responses and leukocyte migration [20, 21]. The components of FhTE have demonstrated significant protective effects on a variety of diseases such as asthma [22], rheumatoid arthritis [21], type 1 diabetes [23], multiple sclerosis [24], and septic shock [25].

In this study, we aimed to substantiate the hygiene hypothesis within the context of MI, providing insights into the potential role of exposure to helminth‐derived antigens in conferring cardioprotection. Using a T2MI rat model induced by ISO, we comprehensively assessed a spectrum of inflammatory and oxidative stress parameters, complemented by electrocardiographic and histological analysis, to investigate whether pretreatment with FhTE could effectively attenuate myocardial injury.

## 2. Materials and Methods

### 2.1. Animals and Animal Care

Male Wistar rats were obtained from the Royan Institute of Biotechnology (Isfahan, Iran) and housed in standard cages, with free access to a pellet diet and water. They were maintained under controlled environmental conditions, with a temperature of 22 ± 1°C and a relative humidity of 45 ± 10%, following a 12‐h light/dark cycle. After a 1‐week acclimatization, the rats, weighing 260 ± 30 g, were randomly assigned to four groups of 10. The sample size (*n* = 10 per group) was determined based on the established practices and outcomes reported in similar in vivo studies of cardioprotection using rodent models [26–28]. All experimental protocols were approved by the Research Ethics Committee of Shahrekord University of Medical Sciences (Ethical Code: IR.SKUMS.AEC.1402.042) and conducted in accordance with the US National Institutes of Health (NIH) Guide for the Care and Use of Laboratory Animals (NIH Publication Number 85‐23, Revised 1996), with measures to minimize suffering, including anesthesia and humane euthanasia via cardiac puncture.

### 2.2. Preparation of FhTE

Adult *F. hepatica* flukes (field isolates from Isfahan, Iran) were collected from naturally infected sheep livers at a local slaughterhouse. The worms were thoroughly washed several times with warm phosphate‐buffered saline (PBS, pH = 7.4) to eliminate contaminants. The identity of the flukes was confirmed through both morphological examination and polymerase chain reaction (PCR) analysis.

About 1 g of the cleaned worms was homogenized in 3 mL of lysis buffer (50 mM Tris pH = 8.0, 2 mM EDTA, 2 mM 2‐mercaptoethanol (2‐ME), 100 mM NaCl, 5% glycerol, and 1× antiprotease cocktail). The homogenate was subjected to centrifugation at 13,000 rpm for 30 min at 4°C to obtain the supernatant containing soluble proteins. The supernatant was dialyzed against 20 mM potassium phosphate buffer (pH = 8.0) to remove low‐molecular‐weight impurities [24]. The dialyzed sample was then concentrated by ultrafiltration using a 4 kDa molecular weight cutoff membrane (Eppendorf, Hamburg, Germany) [29]. The resulting extract was stored as FhTE in 1 mL aliquots at –80°C. The extract was stored at this temperature for up to 1 month. For storage beyond 1 month, new batches were freshly prepared to ensure optimal protein quality and stability.

Sodium dodecyl sulfate–polyacrylamide gel electrophoresis (SDS–PAGE) was performed to verify the homogeneity, purity, and molecular weight profile of the FhTE preparation. Samples (20 µg per lane) from two independent FhTE batches were run on a 15% polyacrylamide gel and visualized with Coomassie Brilliant Blue staining. The total protein concentration was measured using the Bradford Assay [30].

### 2.3. Induction of MI

MI was induced in rats via subcutaneous (s.c.) administration of ISO hydrochloride (Sigma–Aldrich, USA) at a dose of 100 mg/kg, prepared in normal saline (NS, pH = 7.4) to a final volume of 0.3 mL per rat. Injections were delivered at 24‐h intervals over two consecutive days. Animals were euthanized 24 h following the final ISO injection for subsequent analyses [31]. This high‐dose ISO regimen was employed as a model for T2MI‐like ischemic injury because the resulting myocardial damage, driven by β‐adrenergic overstimulation and the subsequent oxygen supply–demand mismatch, closely mimics the nonocclusive pathology of T2MI [32, 33].

### 2.4. Experimental Design

**Figure 1:**
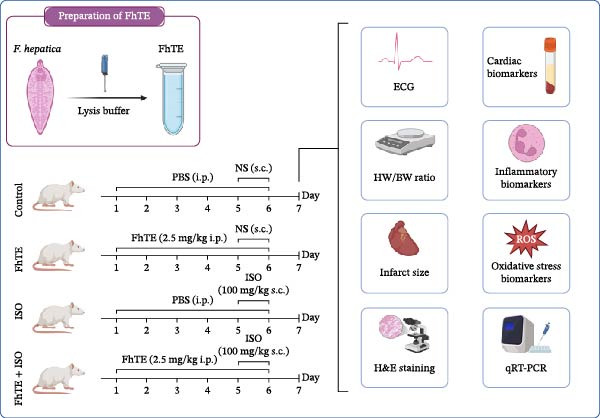
Schematic of experimental workflow and study design (generated with BioRender). *FhTE*: *Fasciola hepatica* total protein extract, *F. hepatica*: *Fasciola hepatica*, *ISO*: isoproterenol, *PBS*: phosphate‐buffered saline, *NS*: normal saline, *ECG*: electrocardiogram, *HW/BW*: Heart weight/body weight, *H&E*: hematoxylin and eosin, *qRT-PCR*: quantitative reverse transcription polymerase chain reaction. Animal experiments were approved under ethical code IR.SKUMS.AEC.1402.042.

FhTE (2.5 mg/kg in 300 μL sterile PBS, pH = 7.4) was administered intraperitoneally (i.p.) to groups 2 and 4 over six consecutive days. The 2.5 mg/kg dose was selected as optimal based on a pilot study, which demonstrated that this dose reduced myocardial infarct size most sizably and mitigated ECG changes most effectively. The pilot data also confirmed the absence of acute toxicity at this dose, as evidenced by stable survival rates and the lack of dose‐dependent adverse ECG changes. Comprehensive details regarding the pilot study are provided in the *Supporting Information*. MI was induced in groups 3 and 4 by s.c. injection of ISO at a dose of 100 mg/kg, administered 30 min following FhTE treatment on the 5th and 6th days. Group 1 (control group) received PBS and NS (Figure 1).

About 24 h after the final treatment, rats were anesthetized with a single i.p. injection of a ketamine (80 mg/kg) and xylazine (8 mg/kg) cocktail. Thereafter, cardiac electrical activity was assessed using electrocardiography. Subsequently, blood samples were collected from each rat under anesthesia, and sera were separated by centrifugation at 3000 rpm for 10 min at 4°C and stored at −80°C until further analysis. All rats were euthanized, and heart tissues were collected for histopathological staining and measurements.

### 2.5. Electrocardiography

Electrocardiography was performed using a PowerLab data acquisition system and LabChart 7 software (ADInstruments, Australia). Rats were placed in a supine position 30 min after anesthetization. Cardiac electrical activity was recorded for 5 min in Lead II configuration via subdermal electrodes at a sampling frequency of 1 kHz and a sensitivity of 10 mm/mV. The key parameters analyzed from the electrocardiograms (ECG) included HR, RR interval, PR interval, QRS duration, QT interval, ST segment changes (≥0.1 mV [34]), and the amplitude of the R wave. HR was calculated using the formula HR = 60/RR interval (in seconds) [35]. QT intervals were corrected according to the modified Bazett’s formula [36]. Successful MI induction was defined by ST‐segment elevation ≥0.1 mV in ISO‐treated rats [37].

### 2.6. Assessment of Cardiac Hypertrophy

About 24 h after the final ISO treatment, the rats were weighed. Under anesthesia, their hearts were excised and rinsed with saline (pH = 7.4) to remove excess blood prior to weighing. The heart weight to body weight (HW/BW) ratio was calculated and expressed in milligrams per gram (mg/g) to assess cardiac hypertrophy.

### 2.7. Histological Analysis

Hearts were excised from the rats after anesthetization and fixed in 10% formalin for 48 h. Following fixation, the apex of the hearts was embedded in paraffin, and sections were prepared as 4 μm thick slices. Hematoxylin and eosin (H&E) staining was performed to assess histological changes [38], including necrosis, inflammatory infiltration, and edema. Histopathological examination was performed by analyzing a minimum of 10 fields per section. The extent of these alterations was quantified using a grading scale of none (–), mild (+), moderate (++), and severe (+++) [39]. Scoring was performed by a single pathologist blinded to the treatment groups. Intra‐observer reliability was confirmed by rescoring all slides at a later time point. The stained sections were visualized and captured using a light microscope (Olympus BX43, Japan) equipped with a camera (Olympus U‐TV0.63XC).

To determine myocardial infarct size, the hearts were excised, and the atria were removed before being frozen at −80°C for 30 min. The frozen hearts were subsequently sectioned into 1 mm slices and incubated in a 1% solution of 2,3,5‐triphenyltetrazolium chloride (TTC, Sigma Aldrich, USA) at 37°C for 20 min in the dark with gentle agitation. Following incubation, the slices were fixed in 10% formalin for 4 h. Viable myocardial tissue stained red, while infarcted regions remained pale. The slices were then digitally photographed, and infarct size was quantified using ImageJ software, expressed as a percentage of the heart tissue [38]. A standardized color thresholding procedure was applied consistently across all images.

### 2.8. Biochemical Analysis

Serum levels of cardiac biomarkers, including cardiac troponin I (cTnI), creatine kinase‐MB (CK‐MB), and lactate dehydrogenase (LDH), were measured to evaluate myocardial injury. cTnI was quantified using an enzyme‐linked immunosorbent assay (ELISA) kit (Roche, Switzerland, Cat. Number 05094798190), and CK‐MB and LDH levels were measured with commercial assay kits (Pars Azmoon, Iran; CK‐MB, Cat. No. 116050; LDH, Cat. Number 122050). All serum analyses were performed in duplicate.

Heart tissue samples were weighed and homogenized to evaluate oxidative stress and inflammation markers. The homogenate was analyzed for superoxide dismutase (SOD), catalase (CAT), myeloperoxidase (MPO), nitric oxide (NO), and malondialdehyde (MDA). MPO and NO levels were quantified using commercial assay kits (Navand Salamat, Iran; MPO, Cat. No. NS‐15062; NO, Cat. Number NS‐15042). All tissue analyses were carried out in duplicate.

SOD activity was measured according to the method of Misra and Fridovich [40]. Briefly, heart tissue was homogenized in 50 mM phosphate buffer (pH = 7) and then centrifuged at 10,000 rpm for 15 min at 4°C. 0.1 mL of the tissue supernatant was mixed with 2.5 mL of 0.05 M sodium carbonate buffer (pH = 10.2), and the reaction was initiated by adding 0.3 mL epinephrine solution. The increase in absorbance was recorded at 480 nm every 30 s for 3 min. Quantification of SOD activity employed a unit definition wherein 1 unit (U) corresponds to the amount of enzyme that yields 50% inhibition of epinephrine autoxidation under the assay conditions. Protein concentration was determined by the Bradford method using a commercial assay kit (Navand Salamat, Iran; Cat. Number NS‐15072), and the final SOD activity was expressed as units per mg of protein (U/mg protein).

CAT activity was measured using the Aebi method [41]. Succinctly, heart tissue was homogenized in 50 mM phosphate buffer (pH = 7) and centrifuged at 10,000 rpm for 15 min at 4°C. The supernatant was collected. In a cuvette, 2 mL of phosphate buffer was mixed with 0.1 mL of the supernatant, and the reaction was initiated by adding 1 mL of 30 mM hydrogen peroxide (H_2_O_2_). The decrease in absorbance at 240 nm was recorded every 30 s for 3 min. Quantification of CAT activity utilized an H_2_O_2_ calibration, with 1 U defined as the amount of enzyme required to decompose 1 μmol of H_2_O_2_ per min under the assay conditions. Protein concentration was measured using a Bradford assay kit (Navand Salamat, Iran; Cat. Number NS‐15072), and CAT activity was expressed as units per mg of protein (U/mg pr).

MDA levels were determined using the thiobarbituric acid reactive substances (TBARS) assay as an index of lipid peroxidation [42]. For this purpose, heart tissue was homogenized in PBS (pH = 7.4) for 2 min and centrifuged at 3000 rpm for 10 min. The supernatant was collected, and cold trichloroacetic acid (TCA) was added to precipitate proteins. A portion of the resulting supernatant was mixed with an equal volume of 0.67% thiobarbituric acid (TBA) and incubated in a boiling water bath for 10 min. After cooling, absorbance was measured at 532 nm. MDA concentration was calculated using a molar extinction coefficient of 1.53 × 10^5^ M^−1^·cm^−1^ and expressed as nmol per mg of protein (nmol/mg pr), with protein concentration determined using a commercial assay kit (Navand Salamat, Iran; Cat. Number NS‐15072).

### 2.9. Quantitative RT‐PCR

Total RNA was extracted from heart tissue using REX solution (Pishgam, Iran) according to the manufacturer’s protocol. RNA concentration and purity were assessed using a NanoDrop spectrophotometer (ND‐2000, Thermo Scientific, USA) by measuring absorbance at 260/280 and 260/230 nm. High‐quality RNA samples exhibited A260/A280 ratios between 1.8 and 2.0 and A260/A230 ratios between 2.0 and 2.2. The average RNA yield per sample was 2.1 ± 0.4 µg (mean ± SEM).

For cDNA synthesis, 1 µg of RNA was reverse transcribed using a cDNA synthesis kit (Pishgam, Iran; Cat. Number 100‐201) following the manufacturer’s instructions. The reverse transcription thermal profile consisted of 25°C for 10 min (primer annealing), 37°C for 50 min (reverse transcription), and 85°C for 5 min (enzyme inactivation), performed on a thermal cycler (ASTEC Gene Atlas G02, Japan).

Quantitative real‐time PCR (qRT‐PCR) was carried out using Master Mix Green Without ROX (Ampliqon, Denmark) on a Rotor‐Gene Q system (Qiagen, Germany) to amplify the target genes. The qPCR reactions included an initial denaturation at 95°C for 10 min, followed by 40 cycles of 95°C for 15 s and 60°C for 60 s. All reactions were run in technical duplicate for each biological replicate. Primer specificity was confirmed by melt‐curve analysis (single peak). Primer efficiencies, determined via standard curves, ranged from 92% to 105% (*R* ^2^ > 0.98).

The relative expression of the target genes was normalized to that of the housekeeping gene, beta‐2‐microglobulin (*B2M*), which has been previously validated for stable expression in rodent cardiac tissue under oxidative stress and inflammatory conditions [43–46]. Expression data were analyzed using the 2^−ΔΔCt^ method, with results presented as fold changes relative to the control group. Primer sequences are provided in the Supporting Information, Table S1.

### 2.10. Statistical Analysis

Statistical analysis was performed using GraphPad Prism. Data are presented as mean ± standard error of the mean (SEM). Data normality was assessed using the Shapiro–Wilk test prior to analysis. Differences between groups were analyzed using one‐way ANOVA, followed by Tukey’s HSD *post hoc* test for pairwise comparisons. A *P*‐value of <0.05 was considered statistically significant.

## 3. Results

### 3.1. FhTE Demonstrates Reproducible Protein Composition by SDS‐PAGE

**Figure 2:**
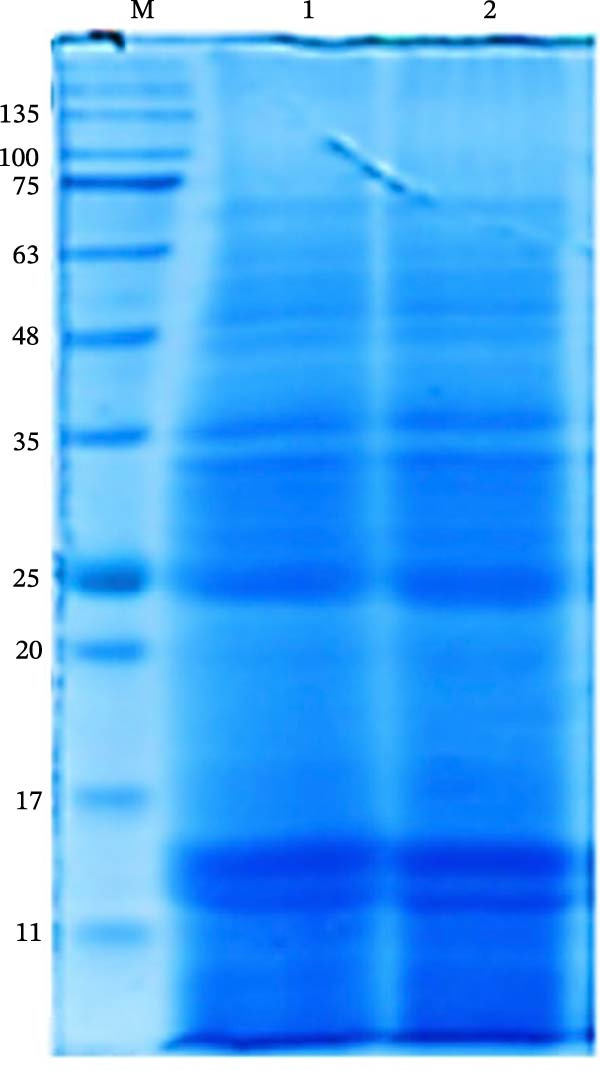
SDS–PAGE profile of FhTE resolved on a 15% polyacrylamide gel and visualized by Coomassie Brilliant Blue staining. Lane M: molecular weight standard (11–245 kDa); lanes 1–2: FhTE preparations from independent batches (20 µg per lane).

SDS‐PAGE analysis of the FhTE preparation revealed highly similar protein profiles across independent batches (Figure 2). Multiple bands spanning ~11–245 kDa were observed, indicating a diverse mixture of proteins. The consistency of banding patterns confirmed the reproducibility of the FhTE preparation and minimal variation in protein composition.

### 3.2. FhTE Reduces Infarct Size, Alleviates Cardiac Hypertrophy, and Attenuates Histological Damage in MI

**Figure 4:**
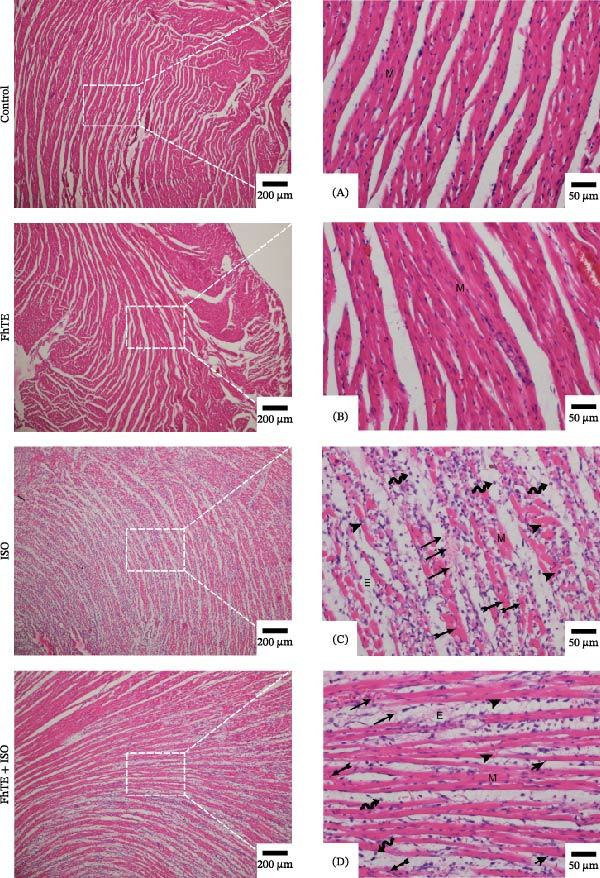
Representative images of apical myocardial tissue stained with hematoxylin and eosin (H&E). (A, B) Intact cardiac muscle fibers with central nuclei and acidophilic cytoplasm (M). (C) Fragmented cardiac muscle fibers with pale cytoplasm (M), vacuolation (arrowhead), inflammatory cell infiltration (wavy arrow), edema (E), karyorrhexis and karyolysis of nuclei (feathered arrow), and extravasated red blood cells (long arrow). (D) Cardiac muscle fibers with less destruction (M), reduced vacuolation (arrowhead), inflammatory cell infiltration (wavy arrow), edema (E), karyorrhexis and karyolysis of nuclei (feathered arrow), and extravasated red blood cells (long arrow). Scale bars: 200 and 50 μm (enlarged views of boxed areas).

Our results demonstrated that FhTE pretreatment significantly reduced infarct size in the MI group, as assessed by TTC staining (Figure 3A). FhTE pretreatment also led to a substantial reduction in cardiac hypertrophy, as evidenced by a significant decrease in the HW/BW ratio (Figure 3B). Moreover, histological analysis with H&E staining revealed that FhTE pretreatment effectively mitigated myocardial necrosis, inflammatory cell infiltration, and edema compared to untreated MI rats (Figure 4 and Table 1), underscoring its cardioprotective potential in the context of myocardial injury. Mortality was minimal across the experimental groups, with a single death observed in the ISO group following the second dose of ISO and no mortality recorded in the control, FhTE, or ISO + FhTE groups.

Figure 3FhTE pretreatment reduces infarct size, cardiac hypertrophy, and cardiac injury biomarkers in the early post‐MI phase. (A) Quantification of infarct size with representative images of heart sections stained with 2,3,5‐triphenyltetrazolium chloride (TTC) (*n* = 5–6 per group). Scale bar: 10 mm. (B) Quantification of heart weight to body weight ratio (*n* = 9–10 per group). (C–E) Quantification of cardiac biomarkers (cTnI, CK‐MB, and LDH) in serum (*n* = 8 per group). Statistical analysis was performed using one‐way ANOVA followed by Tukey’s *post hoc* test. Data are presented as mean ± SEM.

**Figure:**
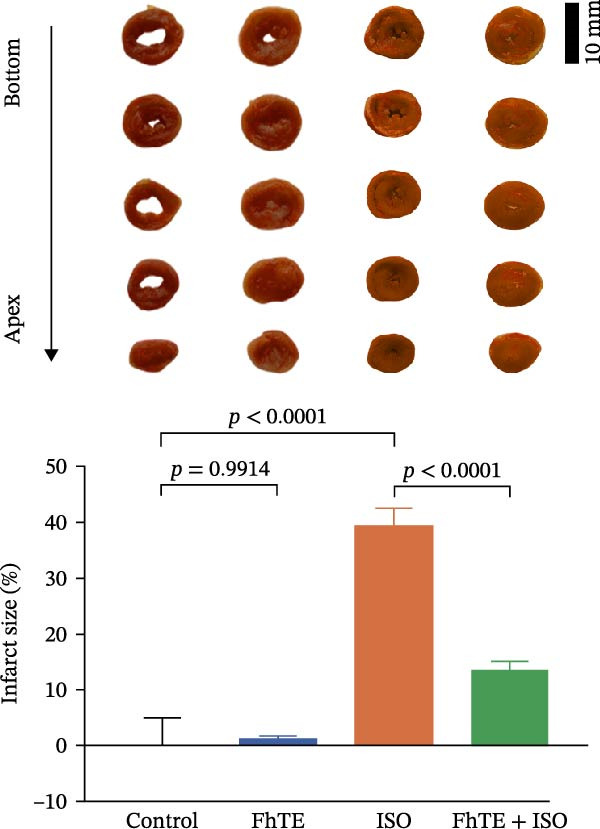
(A)

**Figure:**
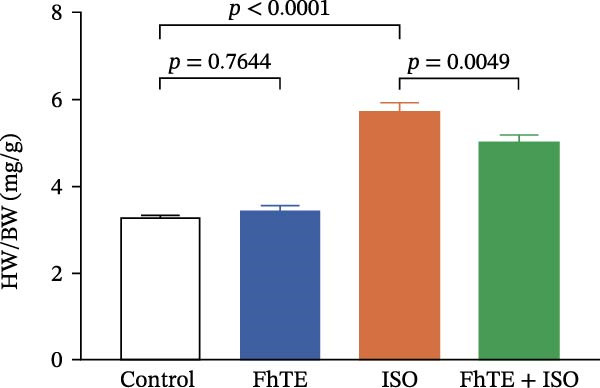
(B)

**Figure:**
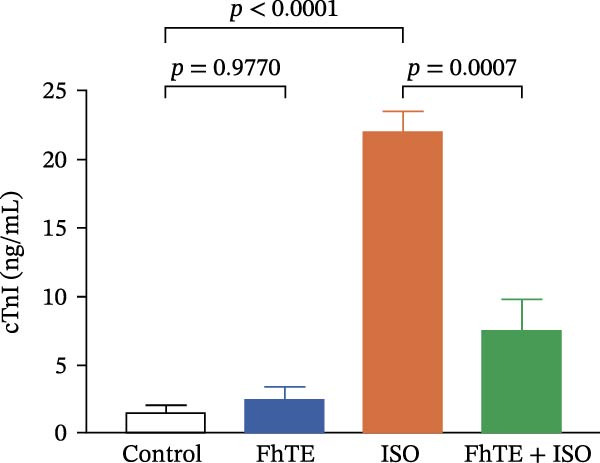
(C)

**Figure:**
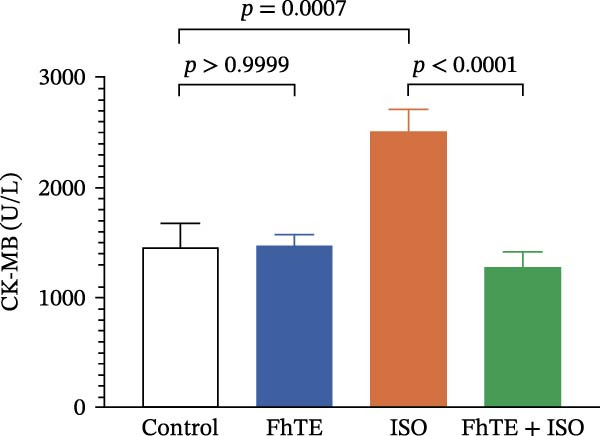
(D)

**Figure:**
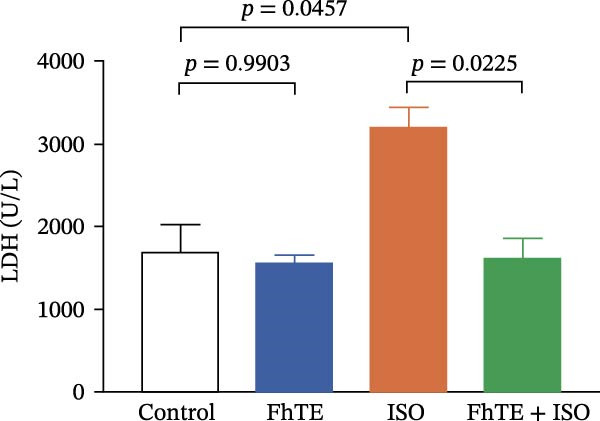
(E)

**Table 1:** Histological alterations in heart tissue on day 7 of the experiment following FhTE pretreatment.

Groups	Necrosis	Inflammatory cell infiltration	Edema
Control	–	–	–
FhTE	–	–	–
ISO	+++	+++	+++
FhTE+ISO	+	++	++

*Note:* Severity is classified as none (–), mild (+), moderate (++), and severe (+++).

### 3.3. FhTE Improves Electrophysiological Parameters by Modulating ECG Changes in MI

ECG analysis showed a significant increase in ST‐segment height in the ISO group compared to the control, thereby confirming the ISO‐induced myocardial injury. Notably, FhTE pretreatment attenuated ST elevation in the FhTE + ISO group, demonstrating its cardioprotective effect. Furthermore, the ISO group exhibited a marked prolongation of the QTc interval, which was significantly reduced following FhTE treatment, suggesting improved electrical stability (Figure 5). The ISO group also displayed a significantly increased HR with a correspondingly shorter RR interval, indicative of tachycardia. FhTE pretreatment effectively reduced HR and prolonged the RR interval in the FhTE + ISO group compared to the ISO group, highlighting its ability to counteract ISO‐induced tachycardia. No significant differences were observed in the PR interval or QRS complex duration between groups. Although R wave amplitude was significantly reduced in the ISO group, no further changes were observed with FhTE pretreatment, indicating that FhTE did not impact the ISO‐induced reduction in R wave amplitude (Table 2).

Figure 5FhTE pretreatment attenuates the electrocardiographic changes associated with myocardial injury. (A) Electrocardiograms (ECG) recorded on day 7 of the experiment, demonstrating shortened RR interval (double‐headed black arrow), ST‐segment elevation (red arrow), and reduced R wave amplitude (purple arrow). (B) Quantification of ST‐segment height and (C) corrected QT interval (QTc) on day 7 of the experiment. Statistical analysis was performed using one‐way ANOVA followed by Tukey’s *post hoc* test. Data are presented as mean ± SEM (*n* = 9–10 per group).

**Figure:**
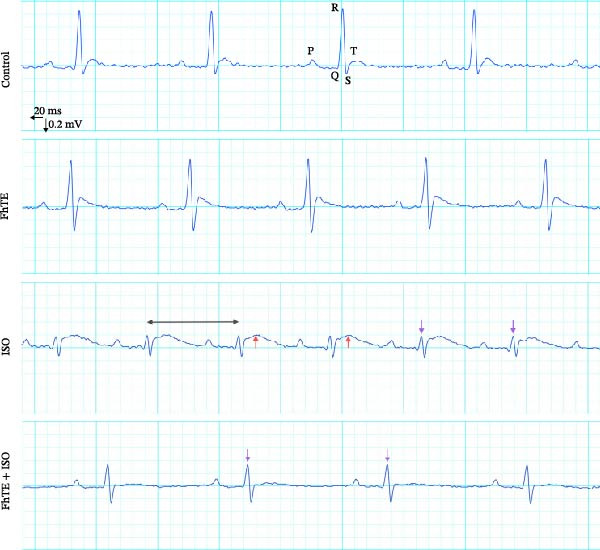
(A)

**Figure:**
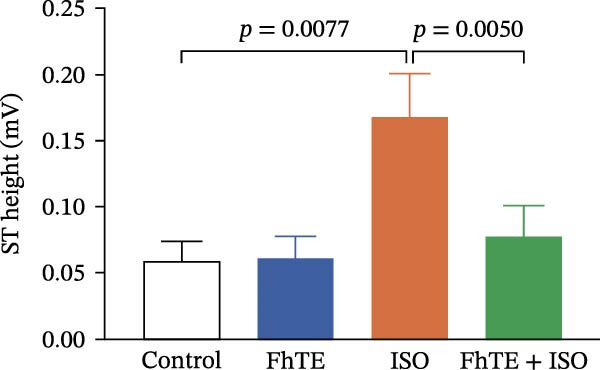
(B)

**Figure:**
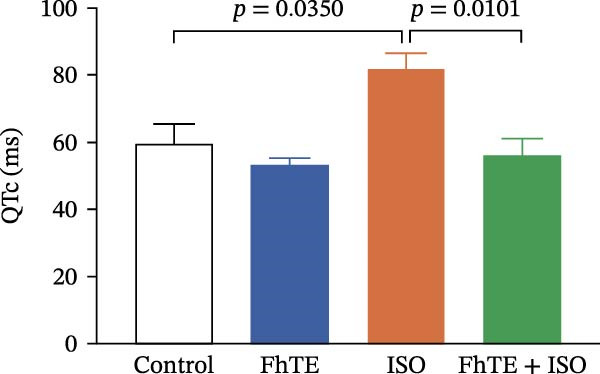
(C)

**Table 2:** Electrocardiographic parameters on day 7 of the experiment following FhTE pretreatment.

Parameters	Control	FhTE	ISO	FhTE + ISO
HR (bpm)	293 ± 8	267 ± 8	424 ± 14 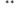	338 ± 22^#^
RR (ms)	206 ± 5.5	227 ± 6.7	142 ± 4.4 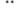	184 ± 11^#^
PR (ms)	52 ± 2.1	51 ± 1.8	46 ± 2.0^ns^	49 ± 1.9^NS^
QRS (ms)	22 ± 2.1	19 ± 0.95	36 ± 9.7^ns^	24 ± 2.2^NS^
*R Amp* (mV)	0.57±0.047	0.57±0.074	0.20 ± 0.019 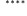	0.21 ± 0.027^NS^

*Note:* Data are presented as mean ± SEM (*n* = 9–10 per group). Statistical analysis was performed using one‐way ANOVA followed by Tukey’s *post hoc* test.

Abbreviations: *NS*, not significant; *ns*, not significant; *R Amp*, R amplitude.

^∗∗^ *p*<0.01.

^∗∗∗∗^ *p* < 0.0001 (vs. Control).

^#^ *p* < 0.05 (vs. ISO group).

### 3.4. FhTE Attenuates Myocardial Injury Biomarkers Associated With MI

Our results demonstrated a significant increase in the levels of cardiac biomarkers, including cTnI, CK‐MB, and LDH, in the ISO group, indicative of substantial myocardial damage. Importantly, pretreatment with FhTE resulted in a marked reduction in the levels of these biomarkers, suggesting a potent cardioprotective effect of FhTE in alleviating ISO‐induced myocardial injury (Figure 3C–E).

### 3.5. FhTE Modulates Neutrophil Activation and Inflammatory Marker Expression in MI

In the context of MI, we evaluated key markers of the primary inflammatory response to determine the effects of FhTE pretreatment. MPO activity, an indicator of neutrophil activation, was significantly elevated in both the FhTE and ISO groups, reflecting an enhanced inflammatory response. ISO administration also resulted in a marked upregulation of mRNA expression for pro‐inflammatory cytokines, including IL‐1β, IL‐6, and TNF‐α, alongside inducible NO synthase (iNOS), a marker of M1 macrophages. Similarly, IL‐33 mRNA levels were significantly increased following ISO administration. Remarkably, pretreatment with FhTE reduced MPO activity and suppressed the mRNA expression of these inflammatory markers (Figure 6), demonstrating its ability to modulate the acute inflammatory response associated with MI.

Figure 6FhTE pretreatment suppresses neutrophil activation and M1 macrophage activity in the early post‐MI phase. (A) Quantification of MPO levels in heart tissue (*n* = 4 per group). (B–F) Relative mRNA expression levels of iNOS and inflammatory cytokines (IL‐1β, IL‐6, TNF‐⍺, and IL‐33) in heart tissue (*n* = 6 per group), normalized to *B2M*. Statistical analysis was performed using one‐way ANOVA followed by Tukey’s *post hoc* test. Data are presented as mean ± SEM.

**Figure:**
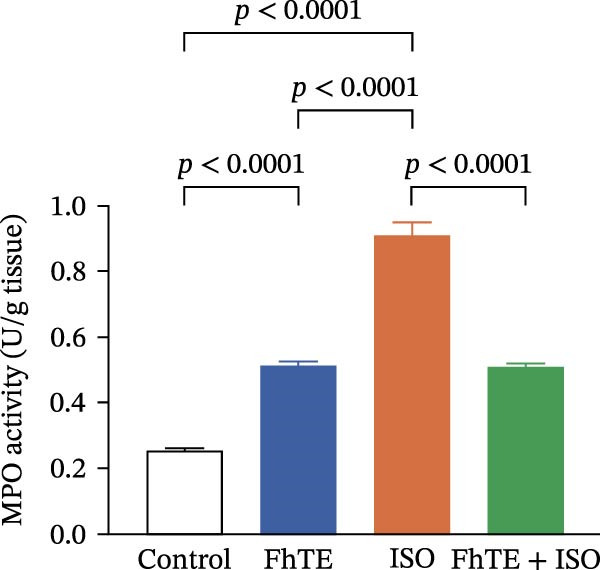
(A)

**Figure:**
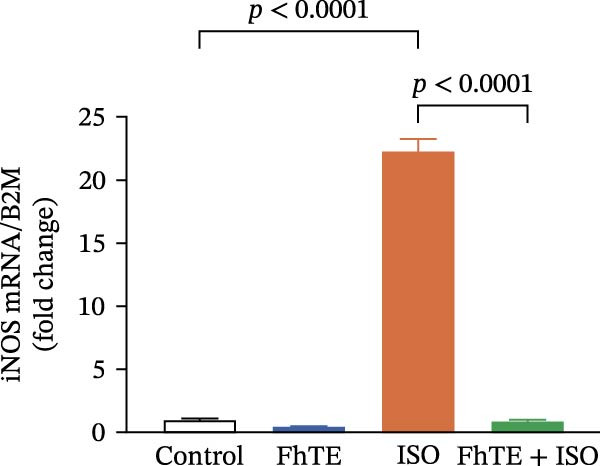
(B)

**Figure:**
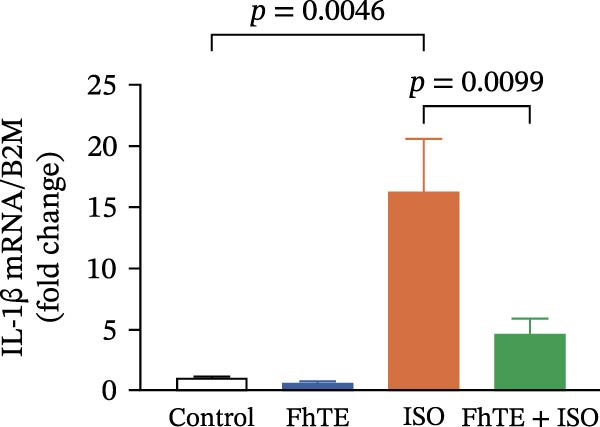
(C)

**Figure:**
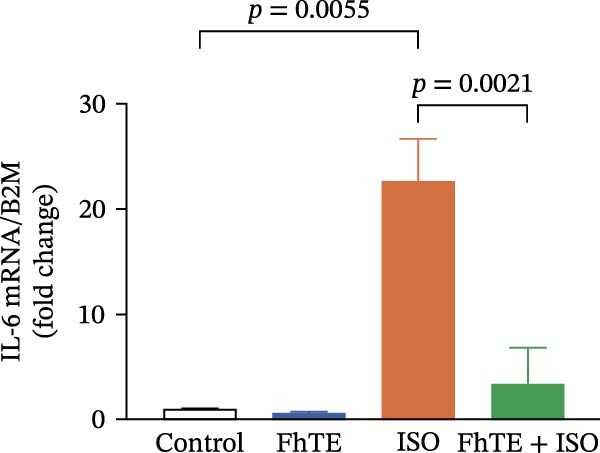
(D)

**Figure:**
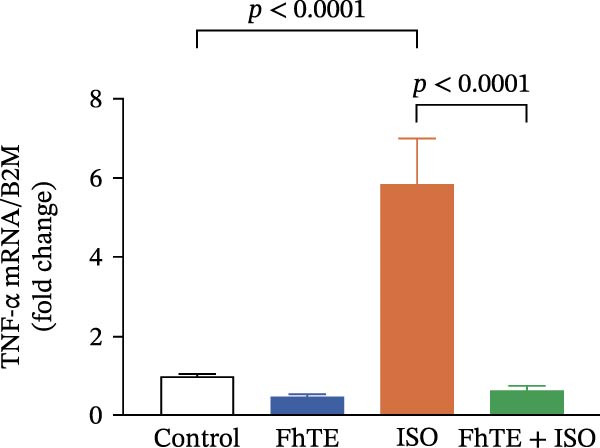
(E)

**Figure:**
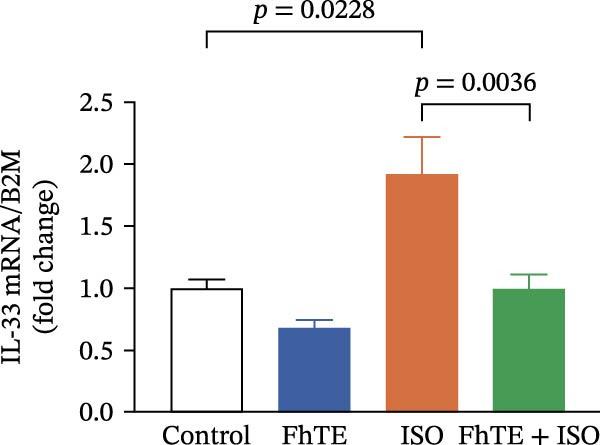
(F)

### 3.6. FhTE Reverses Oxidative Imbalance and Modulates Nrf2 Pathway to Alleviate Oxidative Stress in MI

We assessed the impact of FhTE pretreatment on oxidative stress markers in the context of myocardial injury. In the ISO group, a significant reduction in SOD and CAT activities was observed, accompanied by elevated MDA and NO levels, indicating substantial oxidative stress. Additionally, factor erythroid 2‐related factor 2 (Nrf2) mRNA expression was upregulated in the ISO group, reflecting a compensatory antioxidant response, as this transcription factor regulates cellular antioxidant defenses. In contrast, the FhTE group exhibited decreased SOD and CAT activities, along with increased MDA and NO levels compared to controls, pointing to a potential pro‐oxidant effect of FhTE alone. Notably, pretreatment with FhTE in the ISO group reversed the oxidative imbalance by restoring SOD and CAT activities, decreasing MDA and NO levels, and modulating Nrf2 mRNA expression (Figure 7), thereby effectively mitigating oxidative stress in MI.

Figure 7FhTE pretreatment mitigates redox imbalance in the early post‐MI phase. (A) Relative mRNA expression levels of Nrf2 in heart tissue (*n* = 6 per group), normalized to *B2M*. (B–E) Quantification of SOD, catalase, MDA, and NO levels in heart tissue (*n* = 4 per group). Data are presented as mean ± SEM. Statistical analysis was performed using one‐way ANOVA followed by Tukey’s *post hoc* test.

**Figure:**
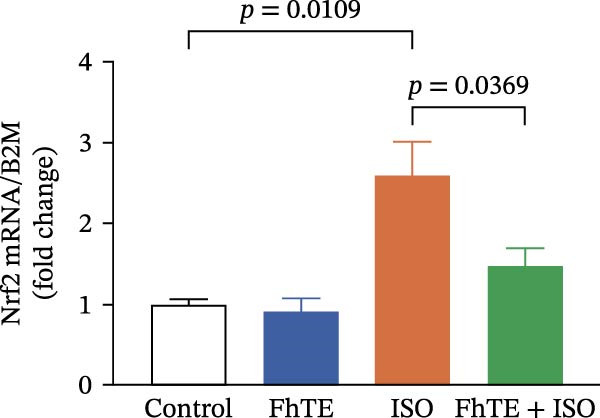
(A)

**Figure:**
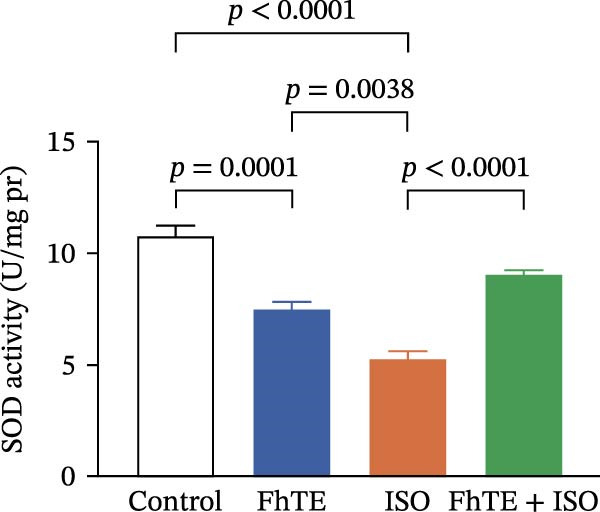
(B)

**Figure:**
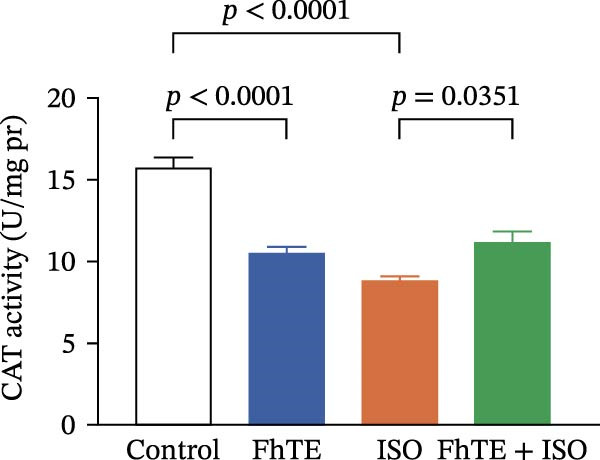
(C)

**Figure:**
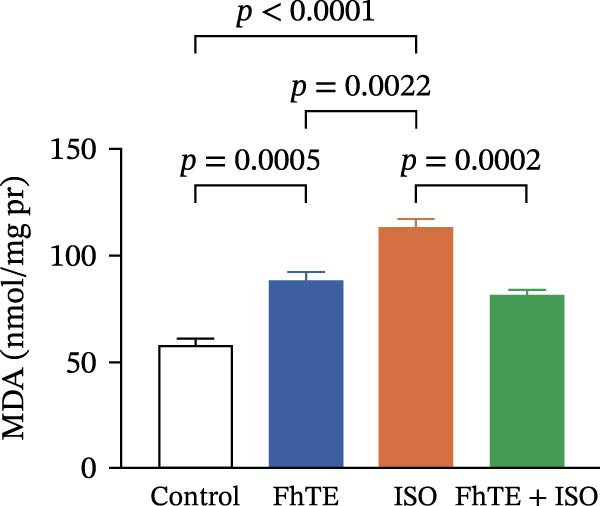
(D)

**Figure:**
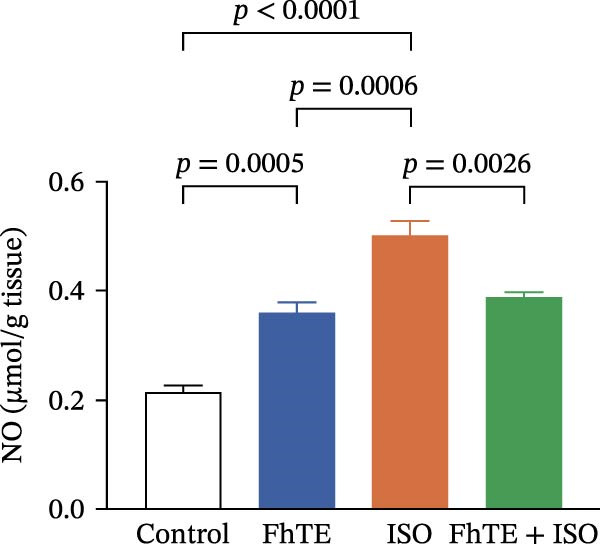
(E)

## 4. Discussion

Emerging evidence highlights the therapeutic potential of helminth‐derived molecules in modulating inflammation, offering innovative avenues for the treatment of cardiovascular diseases such as MI [47]. In this study, we assessed the cardioprotective effects of FhTE in a rat model of ISO‐induced T2MI. To the best of our knowledge, this is the first study to investigate the effects of FhTE in this context. Our results demonstrate that FhTE pretreatment significantly mitigated ISO‐induced myocardial damage, as reflected by reduced infarct size, improved myocardial integrity, and enhanced cardiac electrical stability. Mechanistically, FhTE exerted its protective effects by attenuating MPO activity and inflammatory mediator expression while restoring oxidative balance. These findings suggest that FhTE offers a two‐pronged mechanism, targeting both inflammation and oxidative stress.

The innate immune system plays a pivotal role in the acute inflammatory response following myocardial injury, with neutrophils and macrophages being central to the exacerbation of tissue damage [48]. Following MI, neutrophils are among the first innate immune cells to be swiftly recruited to the ischemic myocardium and aid in the clearance of necrotic tissue. However, excessive infiltration or delayed resolution of neutrophils can exacerbate tissue damage and contribute to the progression of injury [49]. These infiltrating neutrophils release MPO, which catalyzes oxidative reactions, producing tissue‐damaging oxidants like hypochlorous acid [50]. Inhibition of MPO has been demonstrated to reduce inflammation and prevent the progression of cardiac dilation during the early stages of infarct healing [51]. Similarly, cystatin derived from the blood trematode *Schistosoma japonicum* significantly reduced MPO levels in a mouse model of sepsis‐induced cardiomyopathy. This reduction was linked to improved cardiac function and attenuation of inflammation and myocardial injury [52]. In our study, FhTE alone increased MPO activity, whereas pretreatment with FhTE significantly reduced MPO levels in the context of MI. This reduction was linked to a decrease in infarct size, improved ECG parameters, particularly ST elevation, lower levels of cardiac biomarkers (cTnI, CK‐MB, and LDH), and reduced inflammatory cell infiltration. FhTE may confer cardioprotection during MI by initially promoting neutrophil activation, which facilitates inflammation and the clearance of tissue debris. Subsequently, it modulates neutrophil activity and the broader immune response to attenuate oxidative bursts, foster reparative processes, and limit excessive inflammation and secondary tissue damage [53]. Collectively, these results suggest that FhTE pretreatment mitigates myocardial injury and inflammation, likely through the modulation of neutrophil activation, therefore providing promising therapeutic potential in the acute phase of MI.

Subsequent to MI, there is a marked and rapid expansion in the population of cardiac monocytes and macrophages over the ensuing days [54, 55]. Once monocytes migrate into the myocardium, they differentiate into macrophages, acquiring distinct phenotypic characteristics. In the early stages after MI, macrophages primarily exhibit an M1 phenotype, defined by their pro‐inflammatory gene expression profile and activity, driving the initial inflammatory response [56]. This early pro‐inflammatory response is essential for clearing necrotic tissue and initiating the immune cascade. However, excessive activation of inflammatory pathways during this period can exacerbate myocardial injury, underscoring the importance of tightly regulated immune processes to balance tissue defense and damage [57]. Helminth‐based therapeutic strategies aimed at modulating macrophage activity have been proposed to control inflammation and promote tissue repair, as shown in a study in which excretory/secretory products from the nematode *Trichinella spiralis* ameliorated MI through modulation of macrophage function in mice [47]. Building on these findings, we demonstrated that pretreatment with FhTE significantly attenuated the expression of iNOS and key pro‐inflammatory cytokines, including IL‐1β, IL‐6, and TNF‐⍺, all of which are critically involved in the inflammatory response and macrophage activation following myocardial injury.

IL‐33 plays a multifaceted role in MI and its subsequent cardiac remodeling. This cytokine is constitutively expressed in cardiomyocytes and cardiac fibroblasts, where it is released during cellular necrosis under mechanical stress [58], a process that contributes to myocyte hypertrophy and ventricular fibrosis [59]. Pro‐inflammatory cytokines significantly increase IL‐33 expression in these cells [60]. While IL‐33 has been shown to reduce infarct size, mitigate inflammation, and reduce cardiac hypertrophy through ST2 signaling [61], its overall effect on post‐MI recovery is complex. Despite its beneficial effects, IL‐33 also has been demonstrated to exacerbate maladaptive remodeling, contributing to worsened cardiac hypertrophy and an increased risk of ventricular wall rupture [62]. In our study, IL‐33 expression was significantly elevated in the context of MI. Pretreatment with FhTE effectively reduced IL‐33 expression, which was associated with a significant reduction in cardiac hypertrophy. These findings suggest that IL‐33 may act as a pro‐inflammatory cytokine during the early post‐MI phase. While our study focused on the early phase, further investigations are needed to evaluate FhTE’s effects on late‐phase cardiac remodeling and its potential to influence sustained recovery following MI.

Whereas previous studies on helminth‐based therapies in myocardial injury have primarily focused on the inflammatory response [47, 52], our research also examined the modulation of oxidative stress. We found that FhTE, in addition to its effects on inflammation, significantly influenced oxidative stress markers, highlighting its potential as an integrative therapeutic approach for MI.

In the context of MI, excessive ROS production leads to myocardial injury through direct membrane damage, which in turn triggers the release of inflammatory cytokines [63]. This disruption of cellular homeostasis overwhelms antioxidant defenses, as evidenced by significant reductions in SOD and CAT activities, leading to heightened lipid peroxidation and oxidative damage [64]. Although the Nrf2 pathway is activated as a compensatory response to mitigate oxidative stress, its capacity to counterbalance the extensive ROS generation in MI is often insufficient [65]. A clinical manifestation of this imbalance is observed in patients with acute coronary syndromes (ACS), who demonstrate significantly diminished SOD and CAT activity and increased lipid peroxidation [66]. Pretreatment with FhTE restored redox balance by enhancing SOD and CAT activity, leading to decreased lipid peroxidation and a subsequent reduction in MDA levels. This was associated with a significant reduction in infarct size, alleviation of cardiac hypertrophy, cardiac marker levels, and attenuation of necrotic changes in myocardial tissue. FhTE contains antioxidant components such as glutathione S‐transferase (GST) and peroxiredoxin, which play a role in detoxifying reactive metabolites produced by the host [67, 68]. These enzymatic antioxidants may have contributed to a reduction in SOD and CAT activity in the group treated with FhTE alone, while still exerting protective effects against ISO‐induced oxidative stress.

In contrast to ROS‐induced cellular injury, ROS may also function as crucial intracellular signaling molecules that promote cell protection through the activation and expression of antioxidant enzymes. Additionally, low‐level ROS production may serve as a trigger for ischemic preconditioning (IPC), highlighting its role in cardioprotective mechanisms [69]. Similarly, NO plays an essential role in IPC by activating signaling pathways that enhance myocardial resistance to ischemic injury, including the modulation of mitochondrial function and reduction of oxidative stress [70]. Interestingly, in our study, FhTE alone induced a mild increase in ROS (MDA) and NO levels, yet pretreatment with FhTE reduced oxidative stress during ISO‐induced myocardial injury. This seemingly paradoxical effect may reflect a hormetic or preconditioning mechanism, whereby low‐level ROS or NO primes cardiomyocytes to activate endogenous antioxidant defenses and protective signaling pathways, enhancing their resilience to subsequent oxidative insults [71–73]. In this context, the mild elevation in MDA and NO following FhTE monotherapy likely served as the preconditioning stimulus for the myocardium.

This preconditioning effect is mechanistically supported by the observed changes in NOS isoform expression. NO is a key signaling molecule in the myocardium, synthesized through NOS‐dependent and nonenzymatic pathways. Constitutive isoforms, endothelial NOS (eNOS), and neuronal NOS (nNOS) produce low levels of NO under physiological conditions, supporting vascular homeostasis and cardiomyocyte function. In contrast, iNOS is typically undetectable in resting myocardium but is robustly upregulated in response to pro‐inflammatory cytokines, particularly in activated macrophages, during MI, leading to large NO production. Excessive iNOS‐derived NO generates reactive nitrogen species such as peroxynitrite, which damages cellular structures, including proteins, lipids, and DNA, thereby worsening myocardial injury. Similarly, the NOS‐independent pathway is involved in both physiological and pathological conditions of the heart [74]. The results of our study showed that pretreatment with FhTE significantly reduced iNOS expression and consequently decreased NO levels during MI. FhTE alone led to a mild increase in NO production, without affecting iNOS expression. This modest elevation in NO is likely mediated by eNOS or nNOS, further supporting a hormetic or preconditioning effect of FhTE on the myocardium.

Recent investigations characterize FhTE as possessing multiple regulatory functions, e.g., attenuating clinical symptoms in experimental autoimmune encephalomyelitis (EAE) by inducing an anti‐inflammatory phenotype in innate immune cells [75]; inhibiting leukocyte migration to the knee in acute arthritis [21]; and suppressing pro‐inflammatory cytokine and iNOS expression in septic shock through a fatty acid binding protein derived from the extract [76]. The robust cardioprotective effects observed in the ISO‐induced MI model further suggest significant therapeutic potential. FhTE’s ability to simultaneously modulate inflammatory and oxidative responses while triggering an adaptive preconditioning mechanism highlights a potent, multitarget therapeutic strategy. The complexity of FhTE’s numerous immunomodulatory components is a major strength enabling this pleiotropic effect. The next crucial phase involves in‐depth characterization of the active individual components to pave the way for an optimized, standardized therapeutic formulation to preserve cardiac function in IHD and support progression toward future translational research.

Notwithstanding the promising findings, several pertinent limitations warrant consideration. First, the T2MI rat model employed in this study, which exclusively utilized male animals, may not fully encompass the intricate pathophysiology of human MI. This is particularly relevant given the established sex differences in cardiovascular disease and the corresponding variation in immune and inflammatory responses between male and female subjects. Second, although FhTE demonstrated considerable cardioprotective efficacy, the precise bioactive component(s) responsible for these effects remain undetermined, necessitating further mechanistic elucidation. Furthermore, the long‐term ramifications of FhTE on myocardial function, as well as its translational potential in clinical applications, have yet to be thoroughly investigated. Thus, future studies must specifically investigate the efficacy of FhTE in female animals to assess the full translational scope and aim to bridge these gaps, thereby facilitating the advancement of targeted therapeutic interventions.

## 5. Conclusion

Our findings highlight the combined role of FhTE pretreatment in regulating inflammation and mitigating oxidative stress during the early post‐MI phase, offering compelling experimental evidence that supports the hygiene hypothesis in the context of MI. The mild oxidative stress induced by FhTE preconditions the myocardium, enhancing its resilience to ischemic injury. These findings provide solid backing for the potential of the hygiene hypothesis to guide the development of novel therapeutic approaches for MI. However, further research is necessary to investigate the clinical relevance and the molecular mechanisms underlying these effects.

## Author Contributions

**Mohammadreza Ahmadi-Beni:** investigation, formal analysis, writing – original draft. **Kobra Mokhtarian**: resources (FhTE preparation). **Gholam Reza Mobini and Najmeh Salehi-Vanani:** investigation (qRT‐PCR experiments). **Somayeh Najafi-Chaleshtori**: investigation (pilot study, biochemical assays). **Maryam Anjomshoa**: investigation (histopathological evaluation). **Fariba Houshmand**: conceptualization, supervision, writing – review and editing.

## Funding

This work was supported by the Shahrekord University of Medical Sciences (Grant 4325) to Fariba Houshmand.

## Disclosure

All authors have read and approved the final manuscript.

## Conflicts of Interest

The authors declare no conflicts of interest.

## Supporting Information

Additional supporting information can be found online in the Supporting Information section.

## Supporting information


**Supporting Information** The supporting file includes the pilot study conducted to determine the optimal FhTE dose, along with Supporting Table S1, which lists the primer sequences used for qRT‐PCR analysis of IL‐1β, IL‐33, IL‐6, TNF‐⍺, iNOS, Nrf2, and the internal control gene *B2M*.

## Data Availability

The data that support the findings of this study are available from the corresponding author upon reasonable request.
