# Supplementary material for: Fasciola hepatica‐Derived Proteins Shield the Heart From Type 2 Myocardial Infarction in Rats by Modulating Oxidative Stress and Inflammatory Imbalance: Insights Relevant to the Hygiene Hypothesis
Source: Oxid Med Cell Longev. 2026 Mar 13;2026:3759583. doi: 10.1155/omcl/3759583 (PMC13140204; doi:10.1155/omcl/3759583)
Supplement: Supplementary file 1 — Supporting Information The supporting file includes the pilot study conducted to determine the optimal FhTE dose, along with Supporting Table S1, which lists the primer sequences used for qRT‐PCR analysis of IL‐1β, IL‐33, IL‐6, TNF‐⍺, iNOS, Nrf2, and the internal control gene B2M. [file OMCL-2026-3759583-s001.docx]

**SUPPLEMENTARY MATERIAL**

***Fasciola hepatica*-derived proteins shield the heart from type 2 myocardial infarction in rats by modulating oxidative stress and inflammatory imbalance: insights relevant to the hygiene hypothesis**

Mohammadreza Ahmadi-Beni, Kobra Mokhtarian, Gholam Reza Mobini, Somayeh Najafi-Chaleshtori, Najmeh Salehi-Vanani, Maryam Anjomshoa, Fariba Houshmand

**1. SUPPLEMENTARY METHODS**

***Pilot Study***

Prior to the main study, a pilot investigation was conducted to identify the optimal dose of FhTE for assessing its cardioprotective effects in a rat model of ISO-induced MI. Four doses of FhTE (0.25, 0.625, 1.25, and 2.5 mg/kg) were administered intraperitoneally for six consecutive days [1], selected based on preliminary data to balance efficacy and safety [2-5]. Cardioprotective efficacy was evaluated by monitoring ECG parameters and infarct size throughout the study. The findings identified 2.5 mg/kg as the most effective dose, offering significant protection against myocardial injury without observable adverse effects. This dose was subsequently chosen for the main study to ensure robust and reproducible outcomes.

**2. SUPPLEMENTARY TABLES**

**TABLE S1:** Sequences of primers used in qRT-PCR analysis.

| Gene | Forward Primer (5’–3’) | Reverse Primer (5’–3’) |
| --- | --- | --- |
| IL-1β | GAAATGCCACCTTTTGACAGTG | TGGATGCTCTCATCAGGACAG |
| IL-33 | ACTGCTAACAGAATCTTGTGCC | ATCCACACCGTCTCCTGATTG |
| IL-6 | TGCCTTCTTGGGACTGATGTTG | TGTTGTGGGTGGTATCCTCTGT |
| TNF-⍺ | TCTTCTCATTCCTGCTTGTGGC | TGATCTGAGTGTGAGGGTCTGG |
| iNOS | TTTGACCAGAGGACCCAGAG | AAGACCAGAGGCAGCACATC |
| Nrf2 | TTGTAGATGACCATGAGTCGC | TGTCCTGCTGTATGCTGCTTC |
| B2M | CGTGATCTTTCTGGTGCTTGTC | GGAAGTTGGGCTTCCCATTCT |

**REFERENCES FOR SUPPLEMENTARY MATERIAL**

[1] C.M. Finlay, A.M. Stefanska, K.P. Walsh, P.J. Kelly, L. Boon, E.C. Lavelle, P.T. Walsh and K.H. Mills, “Helminth products protect against autoimmunity via innate type 2 cytokines IL-5 and IL-33, which promote eosinophilia,” *Journal of Immunology* 196, no. 2 (2016): 703-714.

[2] M.E. Lund, J. Greer, A. Dixit, R. Alvarado, P. McCauley-Winter, J. To, A. Tanaka, A.T. Hutchinson, M.W. Robinson and A.M. Simpson, “A parasite-derived 68-mer peptide ameliorates autoimmune disease in murine models of Type 1 diabetes and multiple sclerosis,” *Scientific Reports* 6, no. 1 (2016): 37789.

[3] M. Hajizadeh, A.A. Saboor-Yaraghi, A.R. Meamar, M. Khoshmirsafa, E. Razmjou, A. Sadeghipour, Y. Bagheri, F. Sadeghi, N. Jalallou and M.H. Kazemi, “The fatty acid-binding protein (FABP) decreases the clinical signs and modulates immune responses in a mouse model of experimental autoimmune encephalomyelitis (EAE),” *International Immunopharmacology* 96, no. (2021): 107756.

[4] S.M. Quinn, K. Cunningham, M. Raverdeau, R.J. Walsh, L. Curham, A. Malara and K.H. Mills, “Anti-inflammatory trained immunity mediated by helminth products attenuates the induction of T cell-mediated autoimmune disease,” *Frontiers in Immunology* 10, no. (2019): 1109.

[5] P. Janhavi, S. Divyashree, K. Sanjailal and S. Muthukumar, “DoseCal: a virtual calculator for dosage conversion between human and different animal species,” *Archives of Physiology and Biochemistry* 128, no. 2 (2022): 426-430.
